# CFD Investigation on Combined Ventilation System for Multilayer-Caged-Laying Hen Houses

**DOI:** 10.3390/ani14172623

**Published:** 2024-09-09

**Authors:** Changzeng Hu, Lihua Li, Yuchen Jia, Zongkui Xie, Yao Yu, Limin Huo

**Affiliations:** 1 College of Mechanical and Electrical Engineering, Hebei Agricultural University, Baoding 071000, China; changzenghu@163.com (C.H.); jiayuchen1981@163.com (Y.J.); mezkxie@163.com (Z.X.); younger1981@hebau.edu.cn (Y.Y.); 2 Key Laboratory of Broiler/Layer Breeding Facilities Engineering, Ministry of Agriculture and Rural Affairs, Baoding 071000, China; 3 Hebei Provincial Key Laboratory of Livestock and Poultry Breeding Intelligent Equipment and New Energy Utilization, Baoding 071000, China

**Keywords:** CFD, positive-and-negative-pressure-combined ventilation, poultry house, indoor environmental parameters

## Abstract

**Simple Summary:**

The environment of caged-laying hen houses is mainly regulated by ventilation, and the performance of the ventilation system directly affects the welfare and production performance of laying hens. In this study, a combined ventilation system was designed and constructed to improve the microclimate of the house in summer. The practicality of the combined ventilation system was verified for the environmental regulation of the laying hen house, comparing with the traditional negative-pressure ventilation system in terms of airflow pattern, airflow velocity uniformity, temperature regulation variability, and relative humidity distribution status.

**Abstract:**

Mechanical ventilation is an important means of environmental control in multitier laying hen cages. The mainstream ventilation mode currently in use, negative-pressure ventilation (NPV), has the drawbacks of a large temperature difference before and after adjustment and uneven air velocity distribution. To solve these problems, this study designed and analyzed a combined positive and negative-pressure ventilation system for laying hen cages. According to the principle of the conservation of mass to increase the inlet flow in the negative-pressure ventilation system on the basis of the addition of the pressure-wind body-built positive-and-negative-pressure-combined ventilation (PNCV) system, further, computational fluid dynamics (CFD) simulation was performed to analyze the distribution of environmental parameters in the chicken cage zone (CZ) with inlet angles of positive-pressure fans set at 45°, 90°, and 30°. Simulation results showed that the PNCV system increased the average air velocity in the CZ from 0.94 m/s to 1.04 m/s, 1.28 m/s, and 0.99 m/s by actively blowing air into the cage. The maximum temperature difference in the CZ with the PNCV system was 2.91 °C, 1.80 °C, and 3.78 °C, which were all lower than 4.46 °C, the maximum temperature difference in the CZ with the NPV system. Moreover, the relative humidity remained below 80% for the PNCV system and between 80% and 85% for the NPV system. Compared with the NPV system, the PNCV system increased the vertical airflow movement, causing significant cooling and dehumidifying effects. Hence, the proposed system provides an effective new ventilation mode for achieving efficient and accurate environmental control in laying hen cages.

## 1. Introduction

The ventilation system is an important part of the environmental regulation of multi-story three-dimensional facilities for chicken houses [1,2], and a good ventilation system can provide a suitable growing environment for poultry [3]. With the promotion of factory-farming mode and the gradual expansion of farming scale, the importance of the poultry-house ventilation system has become increasingly prominent [4]. Therefore, how to effectively manage and regulate the farming environment has been an important research issue in the field of poultry farming [5].

Airflow pattern is the key to the chicken-house ventilation system, and a good airflow pattern can accelerate the exchange of gases in the chicken house, rapidly discharge exhaust gases from the house, and enhance the cooling efficiency [6]. Currently, most poultry houses in China employ mechanical ventilation for environmental regulation, in which the negative-pressure ventilation (NPV) system is widely employed for poultry houses [7,8,9]. In the latest research, scholars primarily focus on three aspects: adjusting mechanical ventilation systems, building structures, and ventilation patterns. They explore optimal control methods by optimizing gas flow paths and improving ventilation efficiency. Essentially, these efforts involve optimizing and upgrading existing negative-pressure or positive-pressure ventilation systems rather than addressing the inherent drawbacks of current ventilation models. In the traditional negative-pressure ventilation system, the air is drawn into the house through the internal and external pressure difference formed by the fan discharging the air inside the house, forming a longitudinal gas flow path, and utilizing the high air velocity it creates to increase the heat exchange between the airflow and the chickens [10]. The method puts higher requirements on the location of the air inlet and whether the size of the air inlet is reasonable [11], and the distribution of the exhaust fan and the air inlet at both ends of the house creates the longest ventilation path, which ensures that more areas of the chicken coop can be effectively ventilated and exchanged; at the same time, it can lead to the deterioration of uniformity in the environmental parameters of the poultry house [12]. In summer, when high temperatures require the ventilation system to be combined with evaporative cooling pads, the airflow through the evaporative cooling pads exhibits low temperatures and high humidity, resulting in a more pronounced difference between front and rear temperatures [13], and the installation of air intakes on both sides results in more ventilation dead spots [14].

With the rapid development of the modern poultry farming industry, the demands on the environment of poultry production have become higher and higher, and the further refinement of the welfare farming standards has placed higher demands on poultry-house ventilation systems [15,16]. In order to provide farming conditions close to natural free-range farming and improve the uniformity of environmental parameters in the house, many scholars have innovated the ventilation mode around different structures of poultry buildings. Cheng et al. [17] attempted to increase the air velocity in the animal-occupied area by adding ceiling deflectors to direct the downward movement of the airflow entering the house under the traditional negative-pressure ventilation mode to enhance the cooling effectiveness. By optimizing the building structure, the method can avoid the air entering the house through the air guide plate to be directly discharged out of the house by the fan end, which improves the ventilation efficiency in the vertical direction of the chicken cage area. Some scholars consider the large span of the chicken coop and the obvious differences in environmental parameters before and after negative-pressure ventilation regulation: the exhaust fan is designed to be located at the top of the poultry building, and the external air enters the building from the bottom air inlet or air inlet duct, which improves the homogeneity of the environmental regulation by forming a bottom-up ventilation path and accelerates the exhaust gas rate [18,19]. The improved airflow pattern greatly improves the efficiency of environmental regulation [20]. How to make up for the deficiency of existing ventilation methods and solve the problem of the poor control performance of existing ventilation modes is the focus of research.

At present, the research of the combined ventilation mode in the field of chicken-house ventilation systems is still blank. In this study, a positive-and-negative-pressure-combined ventilation (PNCV) system is designed for laying hen houses. Specifically, a positive-pressure fan is added to the negative-pressure ventilation system. The positive-pressure fan actively blows air into the house to increase ventilation efficiency in the vertical direction of the chicken house. The exhaust fan and intake fan work together to form a near-atmospheric pressure environment inside the house, accelerating gas flow. Through the uniformity analysis of air velocity parameters and the spatial distribution of environmental parameters, the practicability of the ventilation model is verified. This approach provides a new model for environmental regulation in factory-farming houses.

## 2. Materials and Methods

### 2.1. Design of the Hen-House Ventilation System

The hen-house structure measures 100 m in length and 15 m in width. The sidewalls are designed to be 4.5 m high, higher than the height of the hen cages. The maximum distance from the ground to the ceiling is 5.3 m. The hen cages measure 1.22 m in length, 1.22 m in width, and 0.4 m in height, arranged in a stacked configuration of 4 layers and 5 columns.

#### 2.1.1. Positive-and-Negative-Pressure-Combined Ventilation Structure

**Figure 1:**
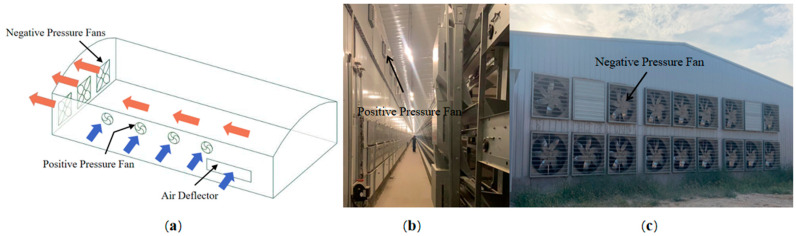
Ventilation structure: (**a**) schematic diagram of the PNCV system structure; (**b**) internal structure of the laying house; (**c**) exterior structure diagram of laying house.

The structure diagram of the positive-and-negative-pressure-combined ventilation (PNCV) system is shown in Figure 1. The negative-pressure fans are installed on one side of the end wall to expel the waste air from the house, creating a negative-pressure environment inside the house. Fresh air from outside enters through the air guide plates. Additionally, positive-pressure fans are installed at the top of the sidewalls, actively supplying air into the house to enhance air circulation.

#### 2.1.2. Configuration of Hen-House Fans

##### Configuration of Negative-Pressure Fans

The design of the hen-house ventilation system is based on the ventilation requirements of the chickens. The internationally accepted standard for calculating ventilation requirements is based on the needs per unit body weight of the chickens. Equation (1) is as follows [21,22]:

(1) $$Q=Q_{s}\times n\times C$$

where Q denotes total ventilation requirement (m^3^/h), $Q_{s}=7m^{3}/h$ denotes maximum ventilation requirement per chicken, n is total number of laying hens, C presents the amplification factor (considering the theoretical calculation and practical deviation, the final result needs to be multiplied by an amplification factor of 1.2 to 1.4).

**Figure 2:**
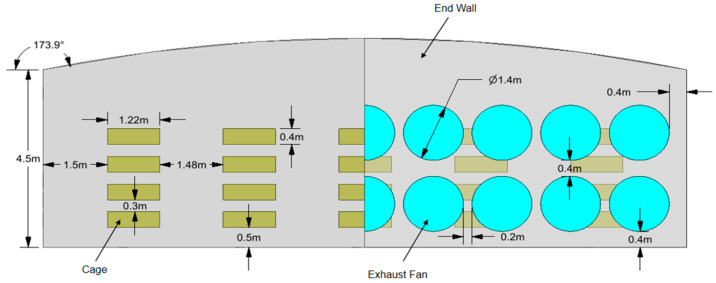
Negative-pressure-fan-structure schematic diagram.

For 42,000 laying hens with an average weight of 1.9 kg, the total ventilation requirement is approximately 660,000 m^3^/h. The design selects exhaust fans based on ventilation requirements, with 18 negative-pressure fans installed in two rows, one above the other, on the dirty side end wall. Each fan has a blade diameter of 1.4 m and a maximum air volume of 38,000 m^3^/h as shown in Figure 2, which can meet the design requirement of completing one air exchange within 1 min [21]. The ratio of exhaust area to intake area should be set as 1:2, with the intake area designed to be 100 m^2^, and the opening angle controlled by hinges [22].

##### Configuration of Positive-Pressure Fans

When the ventilation of the hen house meets the following three assumptions (1) incompressible flow, (2) inviscid flow, (3) steady-state flow, the continuity equation (Equation (2)) can be used to describe the mass conservation in the ventilation system of the hen house.

(2) $$\sum_{i=1}^{n_{in}}A_{i}v_{i}=\sum_{j=1}^{n_{out}}A_{j}v_{j}$$

where $n_{in}$ is the number of inlets, $n_{out}$ is the number of outlets, $A_{i}$ and $A_{j}$ are the cross-sectional areas of the *i*th inlet and the *j*th outlet (m^2^), and $v_{i}$ and $v_{j}$ are the velocities at the *i*th inlet and the *j*th outlet (m/s).

Based on the ventilation volume of the hen house and the effective ventilation area of the air guide plates, the air velocity through the guide plates is calculated to be approximately 5.9 m/s using Equation (2). During the summer, evaporative cooling pads are required for cooling. Excessive inlet air velocity can cause cold stress in the cage area [11]. Hence, a new ventilation design for large-scale poultry houses is proposed to avoid the issue of excessively high or low air velocity in the cage area, namely, the positive-and-negative-pressure-combined ventilation (PNCV) system.

By combining Equation (2) and Bernoulli’s equation (Equation (3)), a comprehensive momentum equation can be obtained as shown in Equation (4).

(3) $$P_{i}+\frac{1}{2}\rho v_{i}^{2}=P_{j}+\frac{1}{2}\rho v_{j}^{2}$$

(4) $$\sum_{i=1}^{n_{in}}P_{i}+\frac{1}{2}{\rho v}_{i}^{2}=\sum_{j=1}^{n_{out}}P_{j}+\frac{1}{2}\rho v_{j}^{2}$$

where $P_{i}$ denotes the pressure on the inner side of the air guide plate (pa), $P_{j}$ denotes pressure on the outer side of the air guide plate (pa). Equation (4) is used to determine the air velocity and pressure distribution across the sections on both sides of the air guide plates [23].

The negative-pressure fans remove air from inside the house, creating a negative-pressure environment. The pressure difference across the air guide plates generates the driving force for air movement, causing fresh air to flow into the hen house at a high velocity through the air inlets. The positive-pressure fans actively supply air into the house, enhancing the internal air circulation and increasing $P_{i}$, which reduces the pressure difference across the air guide plates and, consequently, decreases the inlet air velocity. Considering the total ventilation requirement, the required inlet air velocity, and the inlet area, the positive-pressure fan group needs to provide 324,000 m^3^/h calculated by Equation (2).

To avoid the direct blowing of the fans onto the cage area, the installation height of the fans should be higher than the cage area. The purpose of the positive-pressure fan group is to assist the negative-pressure system in completing ventilation. Due to the overly large inlet area potentially causing excessive local disturbances, fans with a smaller outlet diameter are employed. Fan selection mainly considers the supply air volume, performance curve, fan type and installation size, etc. The supply air volume needs to meet the ventilation needs of the chicken house; different fans have different performance curves under different static pressures. When selecting a fan, ensure that it can provide sufficient air volume under the required static pressure. The fan size must match the actual installation space and environment. After comparing existing fan specifications, fans with a diameter of 0.32 m and an air supply volume of 3250 m^3^/h were selected. According to Equation (5), 95 positive-pressure fans are required. The 95 fans are installed on three walls, with 5 on the front wall, and 45 each on the sidewall.

(5) $$N=Q_{g}/Q_{p}$$

where N is the number of positive-pressure fans, $Q_{g}$ is the ventilation volume required by the positive-pressure fan group (m^3^), and $Q_{p}$ is the ventilation volume of each positive-pressure fan (m^3^).

#### 2.1.3. Structure Design of the Evaporative-Cooling-Pad Room

To improve the cooling efficiency of the ventilation system, this study modified the traditional structure of the evaporative-cooling-pad room. The evaporative cooling pad and ventilation system can be used together to achieve rapid cooling of the laying hen house [24,25]. The configuration ratio of the hen-house volume to the surface area of the evaporative cooling pads was calculated using Equation (6) [26], and the required surface area of the evaporative cooling pads was calculated using Equation (7).

(6) $$\eta_{c}=1-\exp(-\frac{\alpha hL}{\rho vC_{p}})$$

(7) α=A/V

where $\eta_{c}$ is the cooling efficiency of the evaporative cooling pad, v denotes the air velocity through the evaporative cooling pad (m s^−1^), h is the heat-transfer coefficient between the evaporative cooling pad and water (W (m^2^·℃)^−1^), L is the thickness of the evaporative cooling pad (m), $C_{p}$ is the specific heat capacity of air at constant pressure (J (kg·℃)^−1^), and α is the specific surface area of the evaporative cooling pad (m^2^ m^−3^). Additionally,A is the surface area of the evaporative cooling pad (m^2^), and V denotes the volume of the hen house (m^3^).

**Figure 3:**
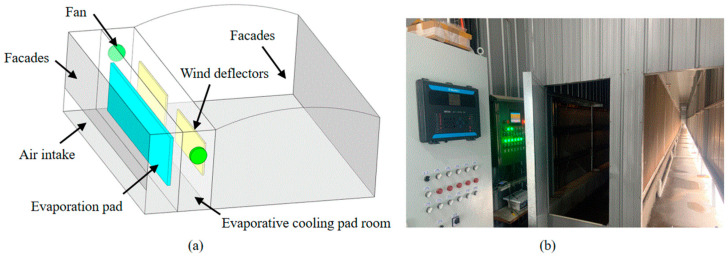
Evaporative cooling pad room: (**a**) structure diagram; (**b**) site structure diagram.

The design of the evaporative cooling pads includes a margin for capacity. Four sets of evaporative cooling pads are installed on each of sidewall A and B, with each set measuring 21 × 2 m. The evaporative cooling pads are installed on the exterior side of the cooling-pad room, as shown in Figure 3. Auxiliary fans are installed inside the cooling-pad room to reduce wind resistance when using the cooling pads and to accelerate the evaporative cooling effect. An enclosure is placed on the exterior side of the cooling pads, with air entering from the bottom opening to prevent the cooling pads from absorbing dust and sand.

### 2.2. Numerical Simulation of Positive-and-Negative-Pressure-Combined Ventilation

A three-dimensional geometric model of the PNCV system was established using SpaceClaim. Fluent was used to simulate the impact of these systems on the spatial distribution of air velocity, temperature, and humidity inside the hen house, assessing the practical effectiveness of the PNCV system [27,28].

#### 2.2.1. Model Setup

**Figure 4:**
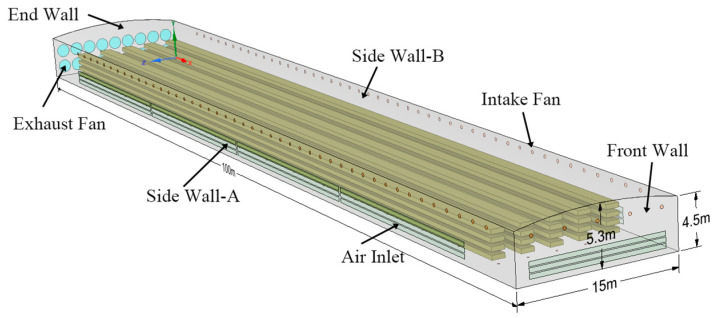
Three-dimensional model of the laying hen house.

In this study, the commercial software Fluent V19.0 was used to perform a three-dimensional simulation of the internal environment of the laying hen house. The geometry of the hen house was modeled using real dimensions (as shown in Figure 4). Large fans were modeled as circles with a diameter of 1.4 m, and small fans were modeled as circles with a diameter of 0.32 m. A coordinate system was established with the ground point at the northeast corner as the origin.

#### 2.2.2. Turbulence Model and Boundary Conditions

To investigate the distribution of environmental parameters under the new ventilation mode, a steady-state analysis was performed, and a pressure-based solver was selected to deal with the complex boundary condition problem. The steady-state analysis assumes that the system remains constant over time, which is very effective for analyzing the ventilation effects and temperature distributions over long periods of operation. Compared to transient analysis, steady-state analysis is more suitable for simulating the average state of the system during stable operation and to quickly obtain the overall performance evaluation of the system, thus providing a reliable basis for design and optimization. For steady-state flow problems, the SIMPLE algorithm was employed to handle coupling between the pressure and velocity fields. To ensure the convergence of the solution, the residual convergence criterion was set to 10^−6^.

Based on the evaluation experiments of three k-ε turbulence models, the RNG k-ε turbulence model, which provides the best predictions for air velocity and temperature–humidity, was selected [27]. The standard wall function (SWF) was used for wall functions.

The gas was set as a mixture of air and water vapor and was considered an incompressible ideal gas, where the gas density depended only on the temperature [18]. First, the airflow speed of a ventilation system is typically low, and the compression effect of the gas can be ignored. Secondly, the ideal gas model can accurately describe the basic thermodynamic properties of air and simplify the calculation. Finally, the model has high computational efficiency, is suitable for large-scale steady-state analysis, and can effectively simulate the temperature and airflow distribution in a laying hen house.

The external air temperature was 32 °C, with a relative humidity of 78%. During the 2 h experimental period, the external air conditions changed minimally. According to the experimental measurements, the inlet temperature and relative humidity remained constant for all application scenarios. Boundary conditions are shown in Table 1.

#### 2.2.3. Porous-Media Zone

The porous-media model was used to simulate the activity area of the laying hens. A rectangular cuboid was drawn according to the actual dimensions, representing the spatial area occupied by the hens. The porous-media zone can be considered as an anisotropic resistance source term as shown in Equation (8).

(8) $$\frac{\Delta P_{r}}{\Delta x_{r}}=-\left.\left\{\sum_{z=1}^{3}D_{rz}\mu v_{z}+\sum_{z=1}^{3}C_{rz}\frac{1}{2}\rho \left|v\right|v_{z}\right.\right\}$$

where $\Delta P_{r}/\Delta x_{r}$ is the pressure drop per unit length for the *r*th (x, y or z) direction (Pa m^−1^), $\left|v\right|$ denotes the magnitude of the velocity (m s^−1^), *D* and *C* are the prescribed matrices for viscous and inertial resistance coefficients, (m^−2^) and (m ^−1^), $v_{z}$ is the inlet air velocity in x, y, or z directions (m s^−1^), μ denotes the dynamic viscosity of air (N s m^−2^), and ρ is the air density (kg m^−3^).

This study uses the geometric shape of the cage, stocking density, and laying hen weight, which are similar to those in Cheng et al. (2018b), to establish the porous medium region based on the data obtained from their experiment [28]. The parameters used are shown in Table 2.

Using a random sampling method, 20 points within the entire hen-house area were selected to measure the body weight of 140 laying hens, and the average body weight was calculated to be approximately 1.9 kg. The heat produced by the hens is transferred to the surrounding environment at a steady rate. During the simulation, the cage area was set as a porous-media region with a fixed heat source. The total heat and sensible heat produced by the laying hens are determined by the following Equations (9) and (10) [29,30]:

(9) $$THP=7m^{0.75}[4\times 10^{-5}{\left(20-T\right)}^{3}+1]$$

(10) $$SHP=[0.8-1.85\times 10^{-7}{\left(T+10\right)}^{4}]\times THP$$

where THP is the total heat production (W), SHP is the sensible heat production (W), T denotes the inlet temperature (°C), and m is the body weight of the hens (kg). Each hen has an average weight of 1.9 kg, and the heat generated in the porous-media region is 338.04 W/m^3^.

#### 2.2.4. Grid Independence Test

**Figure 5:**
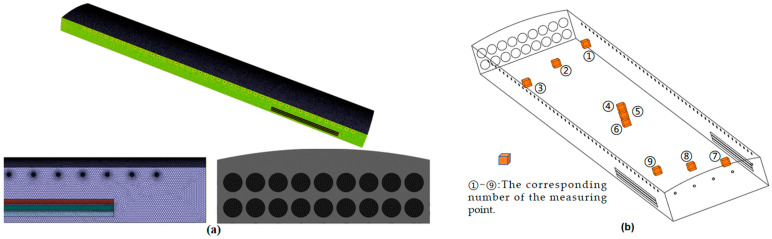
Detailed view of the grid: (**a**) the distribution of the grid; (**b**) the measurement nodes of unrelated solutions.

In this subsection, Fluent Meshing (V19.0) software was used to generate the body mesh, and a combination of tetrahedron–polyhedron was used to divide the grid cells. As shown in Figure 5, fine-mesh grids were used in the areas near the walls, ventilation inlets, and outlets to ensure that the maximum y+ value remained below 5. The gas inside the poultry cage does not require a high-resolution boundary layer; therefore, it was set between 30 and 300 to reduce the computational costs.

**Figure 6:**
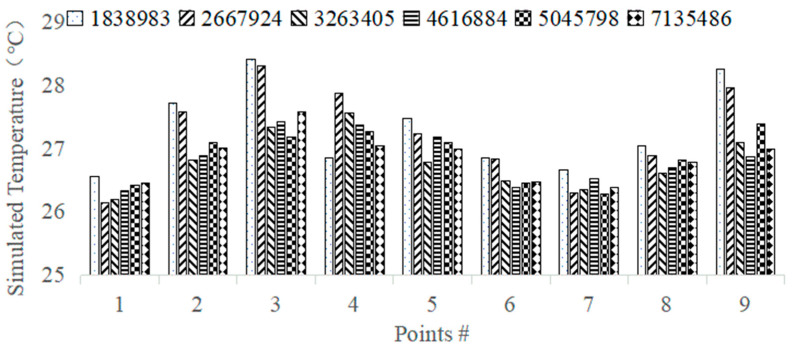
Temperature with different grid sizes.

To reduce the computational burden, the grid specifications were divided into six specifications from one to seven million units for the study of grid-independent solutions, and the air temperatures at nine locations were simulated to compare the accuracy of the simulation results among different grid sizes (Figure 6). The greater the number of grids, the higher the accuracy of calculation results [31]. The computational durations for the 1,838,983, 2,667,924, 3,263,405, 4,222,458, and 5,045,798 grids were 24 min, 46 min, 79 min, 141 min, and 224 min, respectively. In order to balance the progress of the solution and the computational time problem, the calculation results of seven million grid cells are used as the basis to calculate the average relative error with different numbers of grids, which are shown in Table 3. Visibly, the average relative errors corresponding to the 4,222,458 grid and 5,045,798 grid are less than 1% and the difference is not significant; therefore, in order to save the computation time, the 4,222,458 grid is chosen as the computational grid in this study.

#### 2.2.5. Grid Convergence

The grid convergence can be tested during the solving process to determine whether the numerical solution approaches the solution of the real physical phenomenon. In this study, the convergence of the numerical solution was verified by observing the curve of the weighted average temperature on the plane at x = 50 m and by calculating the flux after the computation was completed.

#### 2.2.6. Control Group Experimental Design

**Figure 7:**
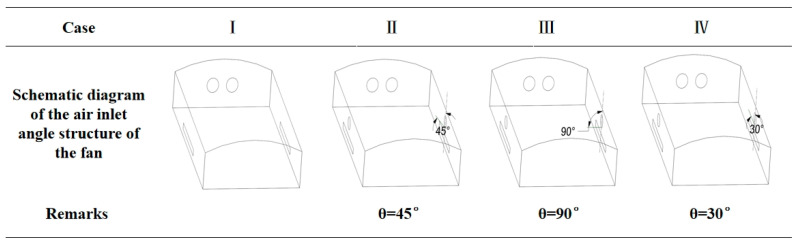
Control group experiment: schematic diagram of fan intake angle.

This study is the first to focus on the combined positive-and-negative-pressure regulation modes to verify the performance of a PNCV system. To facilitate the thorough mixing of fresh outdoor air with the air inside the poultry house, a relatively large empty space is often left under the roof of the laying hen house. Positive-pressure fans were installed at the top of the side walls, positioned higher than the top of the cages. When the positive-pressure fans blew air at an angle no greater than 90°, the airflow did not directly strike the cage area. The ceiling has a certain curvature, and when the positive-pressure fans blow air at different angles, the turbulence intensity and local pressure along the airflow path change, which in turn affects the airflow within the house. To conserve computational resources, the intake angles of the positive-pressure fans were selected as 45°, 90°, and 30° for the experimental conditions. Four experimental schemes were designed for comparison: Case Ⅰ, traditional negative-pressure ventilation system; Case Ⅱ, positive-pressure fan with an angle of 45°; Case Ⅲ, positive-pressure fan with an angle of 90°; and Case Ⅳ, positive-pressure fan with an angle of 30°. As shown in Figure 7, the experiment involved the operation of 14 negative and 95 positive-pressure fans. The ventilation capacity of the negative-pressure fan group was 532,000 m^3^/h, while that of the positive-pressure fan group was 308,750 m^3^/h.

#### 2.2.7. Environmental Analysis Section Planes

**Figure 8:**
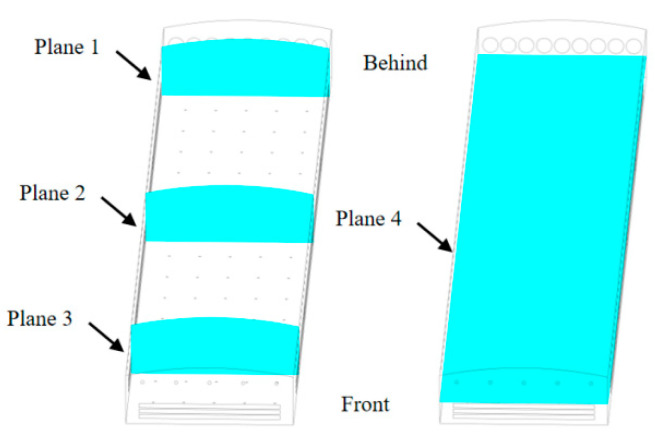
Locations of Planes 1–4 used for the illustration of spatial variations of the indoor environment.

Three vertical planes (Plane 1, Plane 2, Plane 3) and one horizontal plane (Plane 4) were defined within the overall range of the poultry building to illustrate the spatial variations of environmental parameters inside the building. The section planes are shown in Figure 8.

The selection of section planes prioritizes better illustration of the environmental parameters inside the house. According to the building specifications, the planes are divided into three vertical planes, Plane 1, x = 5.495 m; Plane 2, x = 47.495 m; and Plane 3, x = 85.495 m, as well as Plane 4, y = 2 m, which is the height of the third layer of cages.

### 2.3. Measurements

Environmental data measurements were conducted using a self-developed wireless sensor network system, with a Siemens PLC (S7-200 smart, Siemens, Wuxi, China) as the control core, utilizing the Modbus-RTU communication protocol. A LoRa wireless transparent transmission master module (JY-IoT-LoRa, RS485 to LoRa, AMSAMOTION) connected to the PLC. The slave modules were equipped with 9 temperature and humidity sensors (temperature accuracy ±0.5 °C, temperature resolution 0.1 °C; humidity accuracy ±3% RH, humidity resolution 0.1% RH; 485 type) and 9 ultrasonic air velocity sensors (range 0~60 m/s, air velocity ±(0.2 m/s ± 0.02 × v, where v is the actual air velocity), resolution 0.01 m/s, 485 type). All devices were calibrated before measurements.

**Figure 9:**
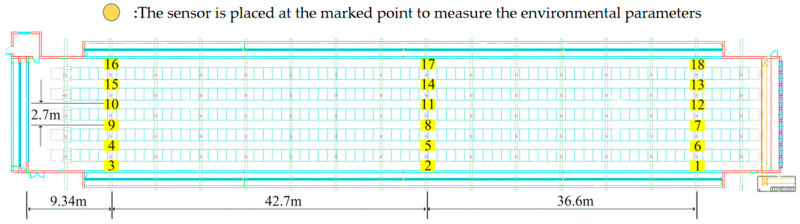
On-site measurement points for environmental parameters.

The CFD model was validated by on-site measurements of environmental parameters under normal farming conditions using the NPV system. Air temperature (T), relative humidity (RH), and air velocity (V) were chosen as reference parameters due to their significant impact on animal thermal comfort [32]. The selection of measuring points is based on the following criteria: (1) Representative areas—we have selected representative areas of the flow field for measurement, including areas where laying hens are active and areas where there may be significant changes in flow velocity. (2) Key position—we set measuring points at the entrance of the flow field in order to accurately capture the performance of the entire system. Measurement points were arranged at two heights: (1) the activity height of the first layer of chickens (0.8 m); (2) the height between the second and third layers of cages (2 m). Eighteen points were selected for each height plane. The diagram of the measurement locations is shown in Figure 9.

The sensors were fixed at 0.8 m and 2 m on a mobile mast, monitoring environmental parameters at a total of 36 points. To ensure the measurement performance of the ultrasonic air velocity sensors, metal deflectors were installed on the mast to keep the sensors in a horizontal position. The mobile mast moved from point 1 to point 18 (as shown in Figure 3b), staying at each position for about 300 s. Fifty samples were collected at each point, and the average was calculated to reduce sample noise [33]. Since the experiment was conducted in a hen house during normal farming operations, to detect any significant changes in the external atmospheric environment during the two-hour measurement period, the three sets of parameters mentioned above were measured at six locations near the air inlet as a reference [31].

### 2.4. Model Validation Method

To validate the accuracy of the hen-house model established in this study, the numerical results of air temperature, relative humidity, and air velocity were compared with the measurement data from 36 points designed on two height planes. To intuitively reflect the differences between the simulation data and the actual measurement data, relative error was used as the validation standard [33]. The formula for relative error is shown in Equation (11). It should be noted that the air velocity values are often small, and using Equation (11) to calculate relative error may result in large relative errors. Therefore, the relative error is usually calculated using the ratio of the simulated difference to the average wind speed at the inlet [34] by modifying the model and repeatedly adjusting the iterative parameters and running multiple simulations to minimize deviations.

(11) $$E_{b}=(\frac{X_{CFD}-X_{EXP}}{X_{EXP}})\times 100\%$$

where $E_{b}$ denotes the relative error, $X_{CFD}$ is the CFD simulation value, and $X_{EXP}$ is the actual measurement value.

Although the CFD model has been verified by the above methods, there are still some limitations. First of all, due to the accuracy limitation of the measuring equipment and the complexity of the field measurement conditions, there may be some errors in the actual measurement data. In addition, certain assumptions and simplifications adopted in CFD models, such as the selection of turbulence models, can lead to deviations between simulation results and reality. To reduce the impact of these limitations, error ranges were taken into account in the data analysis and sensitivity analyses were performed on key parameters to assess the model’s response to different input conditions. Nevertheless, we recommend incorporating more field data and more complex models in future studies to further improve the accuracy of the validation.

### 2.5. Uniformity Analysis of Environmental Parameters

Uniform air velocity distribution within the hen house helps maintain uniform temperature, preventing crowding of the chickens and resulting in drastic environmental changes, thus providing a suitable growth environment. To better evaluate the uniformity of air velocity distribution within a region, this study uses the mass-weighted uniformity index (MWUI) to quantitatively describe the distribution of air velocity in the flow field, as shown in Equation (12). The value of MWUI ranges from 0 to 1, with higher values indicating better uniformity of environmental parameters.

(12) $$\mathrm{MWUI}=1-\frac{1}{\sum_{i=1}^{N}m_{i}}\sum_{i=1}^{N}m_{i}(\frac{v_{i}-\bar{v}}{\bar{v}})$$

where N is the total number of cells in the plane, $m_{i}$ denotes the mass flow rate through the *i*th cell, $v_{i}$ is the air velocity in the *i*th cell, and $\bar{v}$ denotes the mass-weighted average air velocity across the plane.

## 3. Results and Discussion

### 3.1. Model Validation

**Figure 10:**
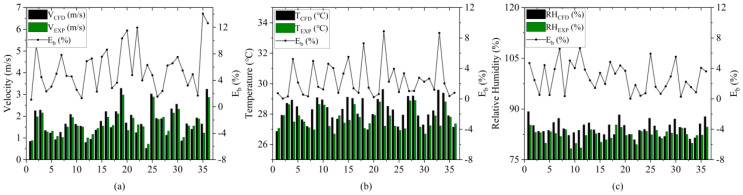
The difference between simulated results and the experimental measurements: (**a**) relative error of air velocity; (**b**) relative error of temperature; (**c**) relative error of relative humidity.

The relative error results of various environmental parameters are shown in Figure 10. As seen in Figure 10, the simulation results of the NPV system exhibit good agreement with the actual measurements. Among the 36 points in the CFD model, 32 points show a relative temperature error of less than 5%, with the predicted air temperature closely matching the experimental measurements. For 31 points, the relative humidity error is below 5%, with the maximum difference being 5.38%, indicating good fitting results. The air velocity differences generally remain within the range of 0.1 to 0.2 m/s. The larger discrepancies are observed at the front end of the poultry house, which can be attributed to the modeling process ignoring automation equipment for egg collection and feeding, as well as construction errors that can lead to measurement inaccuracies. Environmental factors such as temperature, humidity, and gas (such as ammonia) that may exist in the coop may affect the accuracy of the sensor. The positioning of the sensor may also lead to measurement errors. The above errors are within the acceptable range.

The relative error of more than half of the points is less than 5%, and the accuracy of the model can be considered acceptable [6,11]. In this study, by comparing the CFD results of 36 points with the actual measurements, it is concluded that the model of the laying hen house is accurate.

### 3.2. Airflow Patterns

#### 3.2.1. Impact of Positive and Negative-Pressure Ventilation on Air Velocity Distribution in the CZ Area

**Figure 11:**
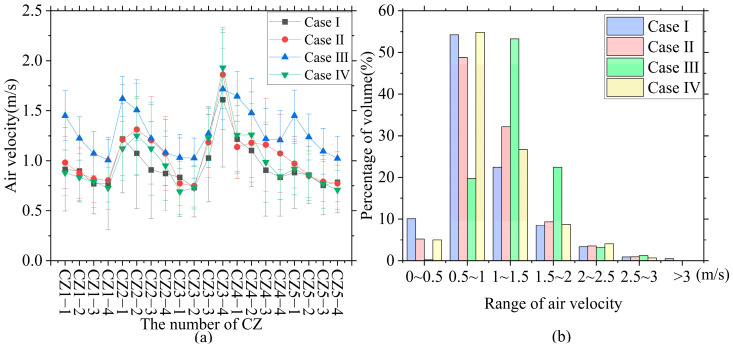
Simulation results of air velocity: (**a**) average air velocity and standard deviation in the CZ; (**b**) proportion of the volume of different wind speeds in CZ.

To facilitate the analysis, the cage zone (CZ) was numbered and expressed as a CZ tier. Figure 11a shows the average air velocity and standard deviation in the CZ. The average air velocity in the NPV system was 0.94 m/s, whereas the average air velocities for the three cases in the PNCV system were 1.04 m/s, 1.28 m/s, and 0.99 m/s, respectively. The proportions of the air velocity volume within different ranges in the CZ are shown in Figure 11b. In the NPV system, 0.5% of the area had an air velocity exceeding 3 m/s, and more than 10% of the area had an air velocity below 0.5 m/s. For the other three cases, the proportions of high air velocity areas (>3 m/s) and low air velocity areas (<0.5 m/s) have decreased, indicating that the PNCV system can reduce ventilation dead zones and decrease the proportion of areas with excessively high air velocity.

**Figure 12:**
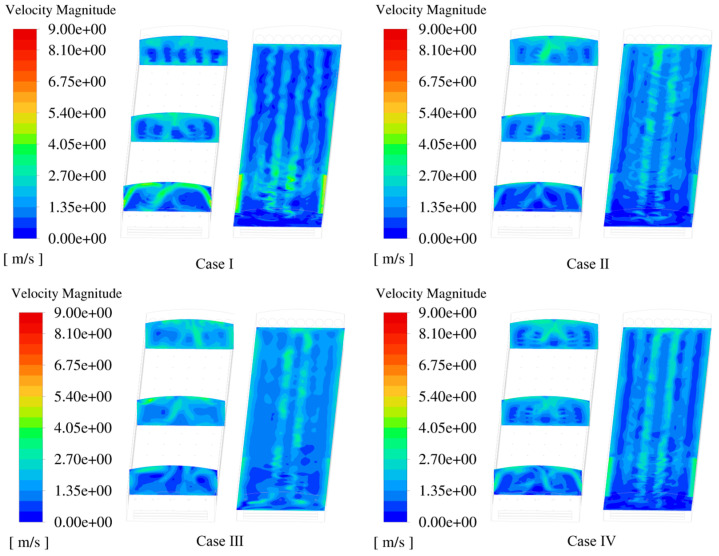
Air velocity distribution in the spatial region.

The airflow distribution pattern inside the building is shown in Figure 12. By actively blowing air, positive-pressure fans increased the vertical airflow within the hen house and improved the average air velocity in the cage zone. By comparing the air velocity distribution contour plots of the X-axis section planes, it can be seen that when the NPV system is in operation, fresh air from the intake openings on both sides of the front end of the hen house is blown diagonally towards the ceiling, forming high-velocity vortices. This results in the highest air velocity in the cage zone at the front end of the hen house reaching 4.45 m/s, which is far above the recommended comfort air velocity of 2.5–3.0 m/s for laying hens in warm climates [30]. When the ventilation system was used in conjunction with evaporative cooling pads, chickens in the core area of the vortex experienced stress reactions to the cold air. In the PNCV system, the positive-pressure fans blow air into the building, merging it with the airflow coming in from the sidewall air guide plates. This results in secondary airflow losses, increases the internal pressure, and reduces the pressure difference across the air guide plates, making the originally high-intake air velocity more moderate. The greater the intake angle of the positive-pressure fans in Cases II, III, and IV, the greater the impact of the air guide plates on the airflow. The maximum air velocities for the three PNCV system schemes were 3.19 m/s, 3.18 m/s, and 3.25 m/s, respectively, and the central aisle maintained a high air velocity, which helps remove more heat and prevents excessive temperatures in the central cage zone.

In the four experiments, excluding the positions of the intake and exhaust openings, the total pressure range varied from −25 to 2 Pa (with atmospheric pressure set to 0 Pa), meeting the welfare farming standards of −25 to −10 Pa [30]. The static pressure environment provided by the PNCV system is closer to natural farming conditions. The pressure differences are shown in Table 4.

#### 3.2.2. Analysis of Air Velocity Uniformity

**Figure 13:**
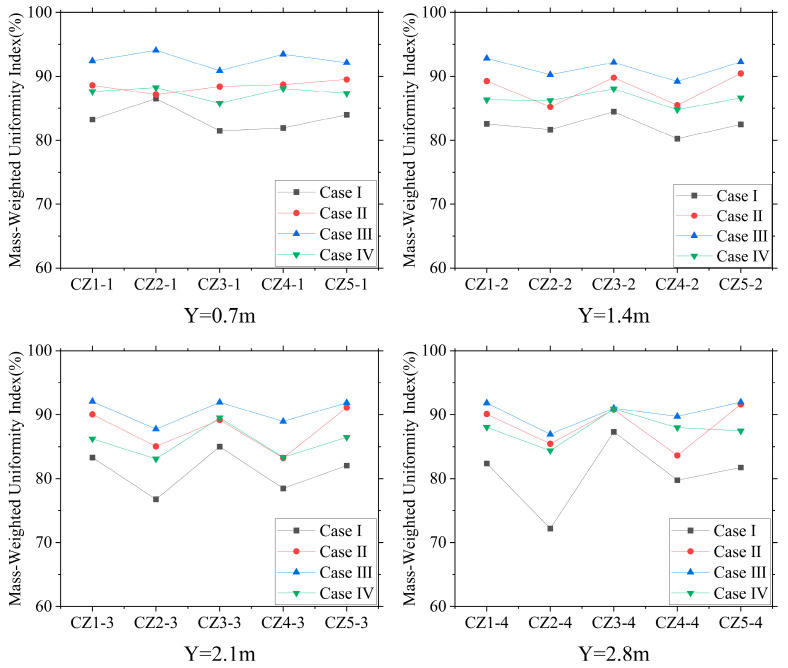
Distribution of mass-weighted uniformity index (MWUI) of air velocity in the CZ.

In this study, the mass-weighted uniformity index of airflow velocity was used to evaluate the uniformity of airflow distribution within the CZ. We examined the mass-weighted uniformity index of the air velocity in the CZ at different heights within the PNCV system (see Figure 13) and compared it with that of a hen house without intake fans.

Figure 13 shows the distribution of the mass-weighted uniformity index of air velocity in the CZ for Cases Ⅰ, Ⅱ, Ⅲ, and Ⅳ within the four-tier hen cage.

This indicates that the uniformity of the air velocity at different heights within the CZs improved in the NPV system with the addition of intake fans. The average MWUI for the three PNCV system cases were 88.14%, 91.18%, and 86.82%, respectively, which were higher than that of the NPV system by 81.88%.

The MWUI in the high-level cage zone for Case Ⅰ showed significant variation, indicating that the intake fans had a more pronounced impact on the vertical airflow movement, thereby improving the effective air velocity in the high-level cage zone. The minimum MWUI for CZ 2-4 in the NPV system was 72.19%. The high intake air velocity from the intake openings on both sides of the building formed strong airflow vortices at high-level cage positions, reducing the uniformity of the air velocity. In the negative-pressure ventilation system, the uniformity decreased with increasing cage height, whereas the PNCV system effectively improved this condition, with Case Ⅲ showing the most significant improvement. This can be attributed to the 90° intake angle of the small fans, which significantly weakened the high-velocity airflow from the intake openings. In Cases Ⅱ and Ⅳ, the intake direction was angled towards the ceiling, with less impact on the incoming airflow. The external air entering the building still formed strong vortices, which affected the uniformity of the indoor air velocity. Adding positive-pressure fans not only increased the average air velocity but also improved the uniformity of the air velocity, demonstrating better ventilation control performance.

### 3.3. Temperature Distribution

**Figure 14:**
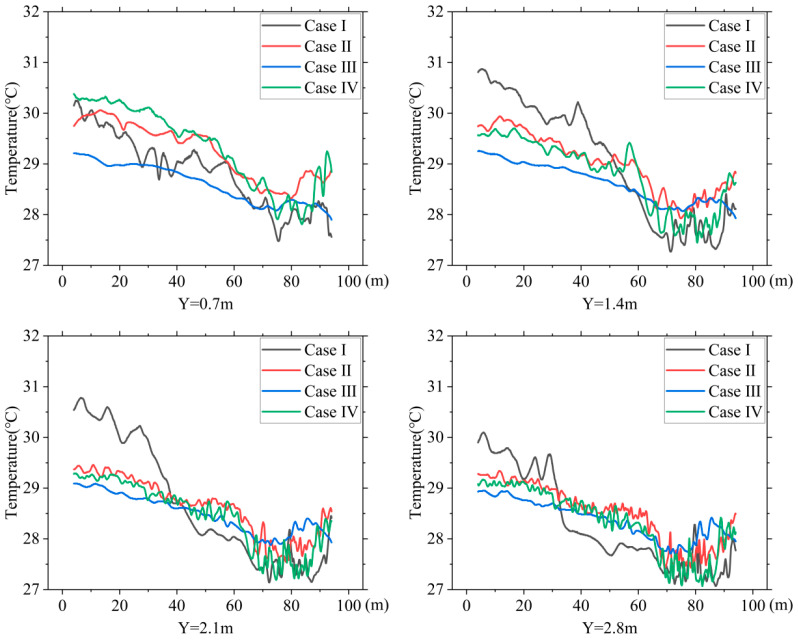
Temperature distribution at the center line of the house for different heights in: Y = 0.7 m, Y = 1.4 m, Y = 2.1 m, and Y = 2.8 m.

Figure 14 shows the temperature variations along the centerline of the building at heights of 0.7 m, 1.4 m, 2.1 m, and 2.8 m (heights of the first, second, third, and fourth tiers of cages) for the four cases. Table 5 summarizes that the maximum temperature difference in the CZ for the NPV system was 4.46 °C, which is higher than the maximum temperature differences of 2.91 °C, 1.80 °C, and 3.78 °C for Cases Ⅱ, Ⅲ, and Ⅳ, respectively.

The PNCV system reduced the temperature difference between the front and rear of the hen house. The NPV system exhibited significant front-to-back differences at all four cage heights. The air cooled by the evaporative cooling pads entered the house from the air guide plates at the front end of the hen house, providing a noticeable cooling effect at the front end. As the cold air moved towards the large fans at the rear end, the cooling effect gradually weakened, and the heat carried by the airflow accumulated at the rear end, causing significant temperature differences between the front and rear areas. The temperature distribution along the X-axis in Case Ⅲ remained relatively constant, staying between 28 and 29 °C. The cooling effects in Cases Ⅱ and Ⅳ were slightly inferior, however, compared with Case Ⅰ, and the temperature differences between the front and rear were significantly reduced.

**Figure 15:**
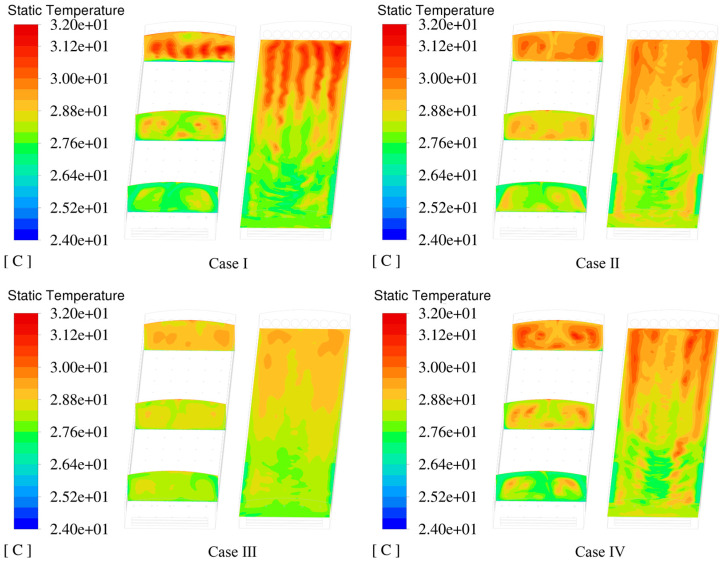
Temperature distribution in the spatial region.

Figure 15 shows the temperature distribution inside the hen house during the summer. When the external ambient temperature reached 32 °C, the temperature in most of the cage areas remained around 30 °C. The temperature at the front end was slightly lower than that at the rear end because the ventilation system created a higher air velocity at the front end, promoting heat dissipation for the poultry.

The X-axis sectional temperature distribution diagram of the NPV system shows the most significant temperature difference between the front and rear areas of the building. The external airflow at the front end entered the interior through the pressure inlet, directly reaching the cage area, increasing the airflow velocity, and enhancing the cooling effect at the front end. The Y-axis sectional temperature distribution diagram shows higher temperatures at the rear end compared with the other schemes. This is because of the negative-pressure mode, in which the indoor air with a higher temperature moves from the front to the rear end under atmospheric pressure, causing excessive heat to accumulate at the rear end of the hen house. When the outdoor temperature is 32 °C in summer, the highest temperature in the cage area is 31.80 °C (Case Ⅰ), and the proportion of the area with temperatures above 30 °C is the largest. The highest temperatures in the cage areas for the PNCV systems Ⅱ, Ⅲ, and Ⅳ were all lower than those in Case Ⅰ, avoiding locally high temperatures. Notably, in Case Ⅲ, with a 90° intake angle, the airflow generated by the positive-pressure fans had the most significant disturbance effect on the airflow entering through the pressure inlet. This reduced the vortex phenomenon at the front end of the building, allowing fresh cold air and the high-temperature indoor air to mix more evenly in the space above the cages, resulting in better cooling effects.

### 3.4. Relative Humidity Distribution

**Figure 16:**
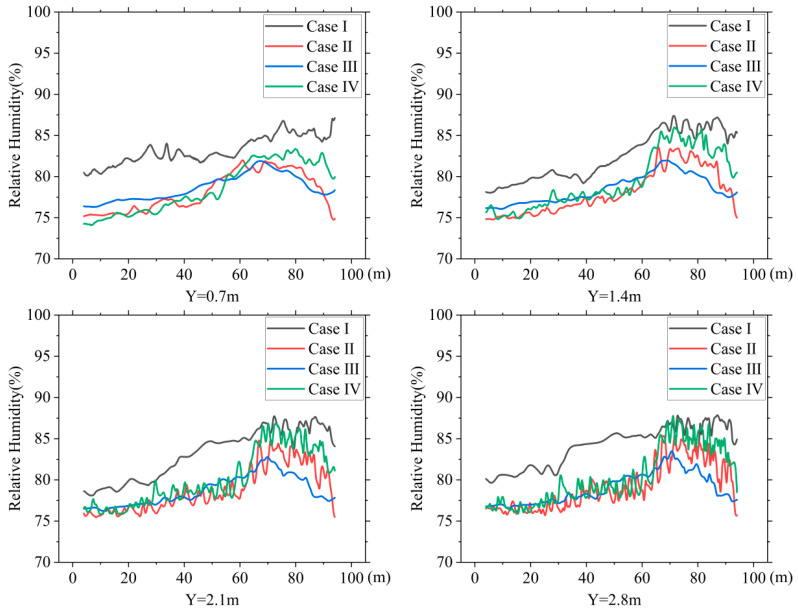
Humidity distribution at the center line of the house for different heights in: Y = 0.7 m, Y = 1.4 m, Y = 2.1 m, and Y = 2.8 m.

Figure 16 shows the relative humidity variations along the centerline of the building at heights of 0.7 m, 1.4 m, 2.1 m, and 2.8 m for the four cases. Overall, the high air velocity provided by the PNCV system discharged more water vapor from the house. The relative humidity in the NPV system ranged between 80% and 85%, while the relative humidity in the PNCV system remained mostly below 80%. Comparing the relative humidity variation curves at the four heights, the higher CZ relative humidity curves for Cases Ⅱ and Ⅳ show more noticeable fluctuations. This is due to the higher water-vapor-content air merging in the ceiling area and irregularly blowing towards the CZ.

**Figure 17:**
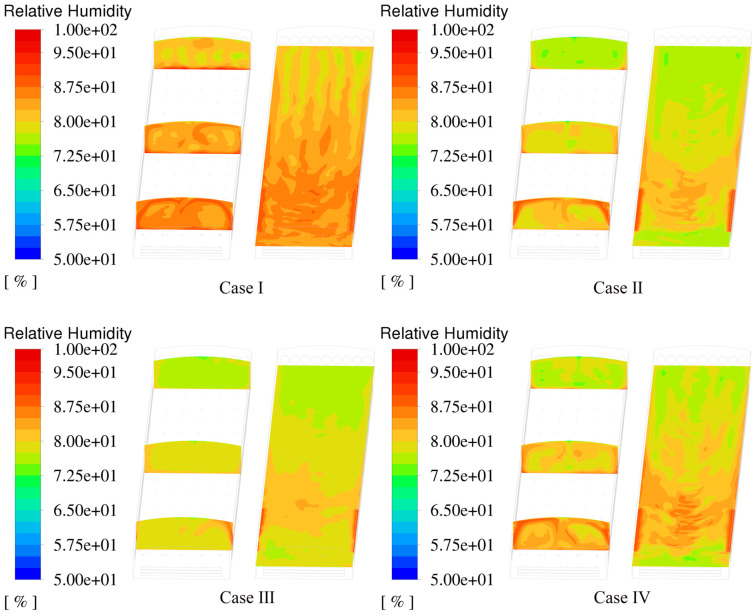
Relative humidity distribution in the spatial region.

Figure 17 shows the relative humidity distribution inside the hen house in summer. Ventilation efficiency directly affects humidity removal within the house. The PNCV system increased the air velocity inside the house, resulting in more noticeable dehumidification. The highest relative humidity in the cage area for Case Ⅰ exceeded 87%, higher than the upper limit for caged-laying hens of 85% [35]. The highest relative humidity in the cage area for the three PNCV system cases was below 85%. Compared to the NPV system, the PNCV system showed better dehumidification performance.

### 3.5. Suitability of Ventilation System

The performance of the supporting equipment of the laying house and the nonlinear change of external climate may cause the results to be different from the expectations. Sensitivity tests can be performed to determine which parameters have a significant impact on the results and to explore why some results may differ from what is expected from previous studies.

Whether the combined ventilation system can maintain an effective ventilation effect under different climatic conditions is the key. The PNCV system aims to improve gas flow, so as to improve the cooling and dehumidification efficiency. In the production, the application of PNCV system improved the gas flow, reduced the morbidity of chickens, reduced the number of vaccines used, and basically maintained the egg production rate above 92%.

During the promotion process, the coop geometry, ventilation system, boundary conditions, and suitability of the physical model must be carefully evaluated, and certain adjustments and validation may be required to ensure that the model remains accurate and reliable in the new environment.

## 4. Conclusions

To address the issues of significant temperature differences between the front and rear areas and uneven air velocity distribution in the existing ventilation systems of laying hen cages, a PNCV mode was proposed. A three-dimensional PNCV simulation system was established to simulate the spatial distributions of various environmental parameters inside a cage. The following conclusions were drawn from the control experiments:

(1) Positive-pressure fans actively blow air into the house, enhancing the overall air circulation in the area. The results were as expected, and the average air velocity in the CZ increased from 0.94 m/s to 1.04 m/s, 1.28 m/s, and 0.99 m/s. The positive-pressure fans also increased the internal pressure, reduced the pressure difference across the air guide plates, prevented an excessively high air velocity at the front CZ, and improved the uniformity of the air velocity distribution in the CZ.

(2) The PNCV system increased the vertical airflow movement, enhanced the overall cooling efficiency, and reduced the temperature control differences between the front and rear of the hen house. The maximum temperature differences in the CZ for Cases Ⅱ, Ⅲ, and Ⅳ were 2.91 °C, 1.80 °C, and 3.78 °C, respectively, all lower than the difference of 4.46 °C in the NPV system.

(3) Positive-pressure fans accelerate air movement, helping to expel more water vapor from the house. The relative humidity in the PNCV system remained mostly below 80%, whereas that in the NPV system remained between 80% and 85%.

The poultry building selected for this study was the first to attempt a combined positive and negative-pressure regulation method with improved ventilation performance and temperature and humidity regulation abilities. Compared to a traditional commercial chicken coop, the density of laying hens in this coop is lower, and the non-occupying space is larger. Therefore, further exploration is required to determine the rationality of the PNCV system configuration under high-density conditions. During the study, only the installation angle of the positive-pressure fan was analyzed, and other application methods of combined positive-and-negative-pressure ventilation were not explored further. Therefore, more experiments should be conducted to explore whether the installation height and installation interval of the positive-pressure fan can improve the environmental adjustment ability of layer house.

## Figures and Tables

**Table 1:** Boundary condition settings.

Items	Boundary Conditions
Air guide plate	Pressure inlet, 26 °C
Positive-pressure fan	Velocity inlet, −6 m/s
Negative-pressure fan	Velocity inlet, 10m/s, 26 °C
Ceiling	No slip wall, 31.8 °C
Ground	No slip wall, 24.2 °C
Front wall	No slip wall, 28.9 °C
End wall	No slip wall, 29.7 °C
Side wall-A	No slip wall, 29.4 °C
Side wall-B	No slip wall, 29.6 °C

**Table 2:** Viscous and inertial resistance coefficients in x, y, and z directions.

X-Direction		Y-Direction		Z-Direction	
D_x_, m^−2^	C_x_, m^−1^	D_y_, m^−2^	C_y_, m^−1^	D_z_, m^−2^	C_z_, m^−1^
11,381.20	0.82	22,005.50	3.23	7121.50	2.27

**Table 3:** Mean relative error table.

**Number of Grids**	1,838,983	2,667,924	3,263,405	4,222,458	5,045,798
**Mean Relative Error**	1.85	1.74	1.05	0.96	0.54

**Table 4:** Indoor static pressure changes of four cases.

Case	Differential Pressure (Pa)	Range (Pa)	Cage Zone Pressure Ave (Pa)
Ⅰ	13.87	−22.14~−8.27	−17.53
Ⅱ	12.10	−13.99~−1.89	−7.66
Ⅲ	11.29	−12.63~−1.34	−6.72
Ⅳ	12.26	−16.07~−3.81	−10.37

**Table 5:** Maximum temperature difference in the CZ.

	CZ Maximum Temperature Difference (℃)
Case Ⅰ	4.46
Case Ⅱ	2.91
Case Ⅲ	1.80
Case Ⅳ	3.78

## Data Availability

The data of this study are available from the corresponding author.
